# Ultrasound-Induced In Situ Self-Assembly of Bimodal Micro/Mesoporous UiO-66-NH_2_ Aerogels for High-Performance Congo Red Capture

**DOI:** 10.3390/gels12090778

**Published:** 2026-08-31

**Authors:** Tian Zhao, Shilin Peng, Yan Wu, Tianhang Wang, Xing Zhang, Zhuoheng Li, Xiangjiang Wu, Ying Chen, Yi Chen

**Affiliations:** School of Materials Science and Engineering, Hunan University of Technology, Zhuzhou 412007, China

**Keywords:** metal–organic framework aerogels, ultrasound-assisted synthesis, hierarchical micro-/mesopores, Congo Red adsorption

## Abstract

Metal–organic framework (MOF) powders possess remarkable adsorption capabilities, yet their practical application is severely hampered by poor processability, difficult recovery, and high mass-transfer resistance. Here, we report a green and rapid two-stage strategy for constructing UiO-66-NH_2_ self-assembled aerogels with a bimodal micro-/mesoporous architecture. A brief thermal pretreatment (130 °C, 3 h) is used solely for precursor activation, after which the critical MOF crystallization and in situ self-assembly are driven under ambient conditions via ultrasonic cavitation (900 W, 15–60 min). This protocol simultaneously drives the nucleation, crystallization, and self-assembly of UiO-66-NH_2_ nanocrystals into a monolithic, self-supporting architecture, thereby replacing the conventional prolonged high-temperature solvothermal MOF crystallization with a rapid room-temperature process. The sonication time critically governs the structural evolution, transforming initially amorphous aggregates into well-defined regular octahedral nanocrystals that form an interconnected framework. The optimized aerogel (UNA-T60) exhibits an exceptional specific surface area (1196.9 m^2^ g^−1^) and a synergistic bimodal pore structure comprising intrinsic micropores and intercrystalline mesopores. This architecture enables rapid mass transfer and active-site accessibility, resulting in a maximum Congo Red (CR) adsorption capacity of 660.56 mg g^−1^, with kinetics conforming to the pseudo-second-order model (R^2^ > 0.999). The robust monolithic structure endows the material with outstanding reusability, retaining 90.8% of its initial adsorption capacity after four regeneration cycles. This work presents a paradigm-shifting approach for the sustainable fabrication of pure MOF aerogels, offering a promising solution for advanced dye wastewater treatment.

## 1. Introduction

The accelerating pace of industrialization has rendered water pollution an increasingly severe environmental challenge, with synthetic dyes representing a prominent class of typical contaminants [1,2]. These substances exhibit high chemical stability and resistance to biodegradation; upon entering aquatic environments, they pose long-term hazards to both ecosystems and human health [3,4]. Among synthetic dyes, azo dyes constitute the largest proportion, wherein the azo bond (–N=N–) imparts vivid chromophoric properties, accounting for approximately 60–70% of global annual dye production, with an estimated 10–15% discharged directly into water bodies during manufacturing processes [5,6]. CR, C^32^H_22_N_6_Na_2_O_6_S_2_, serves as a paradigmatic representative of this dye class. It not only exhibits potential dermal sensitization and carcinogenic risks but also demonstrates exceptionally high aqueous solubility attributable to the presence of two sulfonic acid groups (–SO_3_^−^) within its molecular structure [7,8]. Conventional treatment methodologies, including biodegradation, photocatalysis, and chemical coagulation, have proven inadequate for its effective removal [9,10]. Consequently, the development of novel materials characterized by high adsorption capacity, rapid reaction kinetics, and environmental compatibility has become an urgent imperative for the treatment of CR-containing wastewater [11].

MOFs represent a class of porous crystalline materials constructed from the coordination assembly of metal nodes and organic linkers, exhibiting exceptionally high specific surface areas and tunable pore architectures [12,13]. These materials have found extensive applications in gas separation [14], catalysis [15], and pollutant adsorption [16], among other fields. Among them, zirconium-based UiO-66-NH_2_ has garnered considerable attention owing to its robust structural stability and readily accessible synthetic precursors. Its framework is composed of zirconium oxo clusters (Zr_6_O_4_(OH)_4_^12+^) interconnected by 2-aminoterephthalic acid ligands. The amino-functionalized framework of UiO-66-NH_2_ provides a high density of polar surface sites [17] that may contribute to adsorption affinity toward anionic dyes [18]. However, in the absence of direct spectroscopic evidence (e.g., in situ FTIR, XPS, or zeta-potential titration), the present study does not invoke specific interaction mechanisms, such as electrostatic attraction or hydrogen bonding, as explanatory factors. Instead, our analysis focuses on experimentally verified structure–performance relationships, specifically the role of hierarchical micro-/mesoporous architecture and specific surface area in governing adsorption capacity and kinetics. Nevertheless, UiO-66-NH_2_ is predominantly synthesized via solvothermal methods, yielding nanoscale powders that present significant practical limitations: the particulate nature predisposes the material to aggregation, thereby diminishing the accessibility of active adsorption sites; the powder morphology complicates separation and recovery from aqueous media; column packing engenders high hydraulic resistance and susceptibility to clogging; and fine particulate matter may leach into the treated effluent, causing secondary contamination [19,20]. These inherent drawbacks pose substantial barriers to the transition of UiO-66-NH_2_ from laboratory-scale research to engineering-scale applications [21].

To circumvent the engineering limitations of MOF powders, researchers have developed two distinct shaping strategies. The first approach involves substrate-supported composite materials, wherein pre-synthesized MOF crystals are immobilized onto assembly substrates such as cellulose nanofibrils [22], graphene oxide [23], or polymer membranes [24] via impregnation or chemical bonding. Although this methodology improves the mechanical processability and operational convenience of the materials, the introduction of extraneous matrices inevitably dilutes the active MOF component fraction and creates mass transfer barriers at the MOF–substrate interface, resulting in a significant reduction in adsorption capacity compared to pristine MOFs [25]. The second approach entails self-assembled MOF monolithic materials, wherein the interfacial self-assembly behavior of MOF nanocrystals is precisely modulated to directly construct three-dimensional macroscopic frameworks with interconnected porosity [26,27]. Such pure MOF aerogels retain the full active material density while simultaneously establishing a micro-/mesoporous network: micropores (<2 nm) originating from the intrinsic MOF pore channels provide abundant surface adsorption sites, whereas mesopores (2–50 nm) arising from the interstitial packing gaps between nanocrystals facilitate the diffusion of dye molecules into the interior [28,29]. The synergistic interplay among these hierarchical pore levels endows the material with both high specific surface area and low mass transfer resistance, thereby achieving concurrent optimization of adsorption capacity and kinetics [30,31]. Although significant progress has been made in the construction of MOF aerogels in recent years, existing methodologies generally rely on high-temperature solvothermal conditions (>100 °C) and toxic solvents such as N, N-dimethylacetamide (DMAc), accompanied by protracted aging periods. These requirements present pronounced deficiencies in terms of energy consumption, operational safety, and environmental footprint [32].

To facilitate the transition toward green and mild preparation protocols, room-temperature synthesis strategies offer a viable pathway. However, intrinsic thermodynamic limitations—insufficient nucleation driving force and sluggish growth kinetics—necessitate external energy input, as static conditions alone are inadequate for obtaining well-crystallized products or driving nanocrystal assembly into three-dimensional networks [33]. Ultrasound-assisted synthesis precisely addresses this dual requirement. The underlying physical mechanism resides in the cavitation effect induced by ultrasonic wave propagation through the liquid phase: cavitation bubbles nucleate and expand during the negative pressure phase of the acoustic cycle, followed by violent collapse during the positive pressure phase. At the moment of collapse, localized extreme conditions of approximately 5000 K, ~1000 bar, and cooling rates exceeding 10^10^ K s^−1^ can be generated [34]. Such extreme non-equilibrium microenvironments of rapid heating and quenching effectively reduce the crystal nucleation energy barrier, while the ultrafast quenching effect suppresses Ostwald ripening of crystallites, yielding nanocrystals with uniform dimensions and low defect densities [35]. The accompanying microjetting and turbulent perturbation enhance mass transfer and surface cleaning, improving reaction uniformity [36,37]. Importantly, the stochastic aggregation of nanocrystals within the ultrasonic field disrupts thermodynamically controlled assembly, creating a unique kinetic window for in situ generation of mesopores [38,39]. Nevertheless, existing ultrasound-assisted room-temperature syntheses have been confined to MOF nanopowders, and extending this methodology to micro-/mesoporous pure MOF aerogels requires elucidating nanocrystal assembly behavior, pore evolution mechanisms, and gel skeleton stabilization under ambient conditions [40,41].

Based on the aforementioned analysis, this work proposes a two-stage ultrasound-assisted strategy for the ambient-temperature crystallization and in situ self-assembly of micro-/mesoporous UiO-66-NH_2_ aerogels, entirely without extraneous binders or polymeric substrates. Stage I employs a brief thermal pretreatment (130 °C, 3 h) solely for precursor activation; Stage II drives the subsequent MOF nucleation, crystallization, and self-assembly by ultrasonic cavitation at room temperature and pressure. By modulating precursor stoichiometry, solvent composition, and ultrasonic parameters, rapid nucleation and spontaneous assembly of UiO-66-NH_2_ nanocrystals are achieved, inducing an in situ sol–gel transition to yield three-dimensional monolithic aerogels with an interconnected micro-/mesoporous network [42,43]. To validate the feasibility of this strategy, the aerogels were systematically characterized with respect to crystal structure, pore characteristics, surface chemistry, and mechanical stability. Their adsorption performance toward CR solutions was investigated under ambient conditions, and isotherm models were employed to analyze the adsorption behavior and capacity characteristics [44,45]. Furthermore, reusability and practical application potential were evaluated through cyclic regeneration experiments [46]. This work not only provides a novel approach for the green and rapid fabrication of pure MOF aerogels via a room-temperature ultrasound-driven process but also opens new avenues for the rational design of micro-/mesoporous adsorbent materials and their application in dye wastewater treatment.

**Scheme 1:**
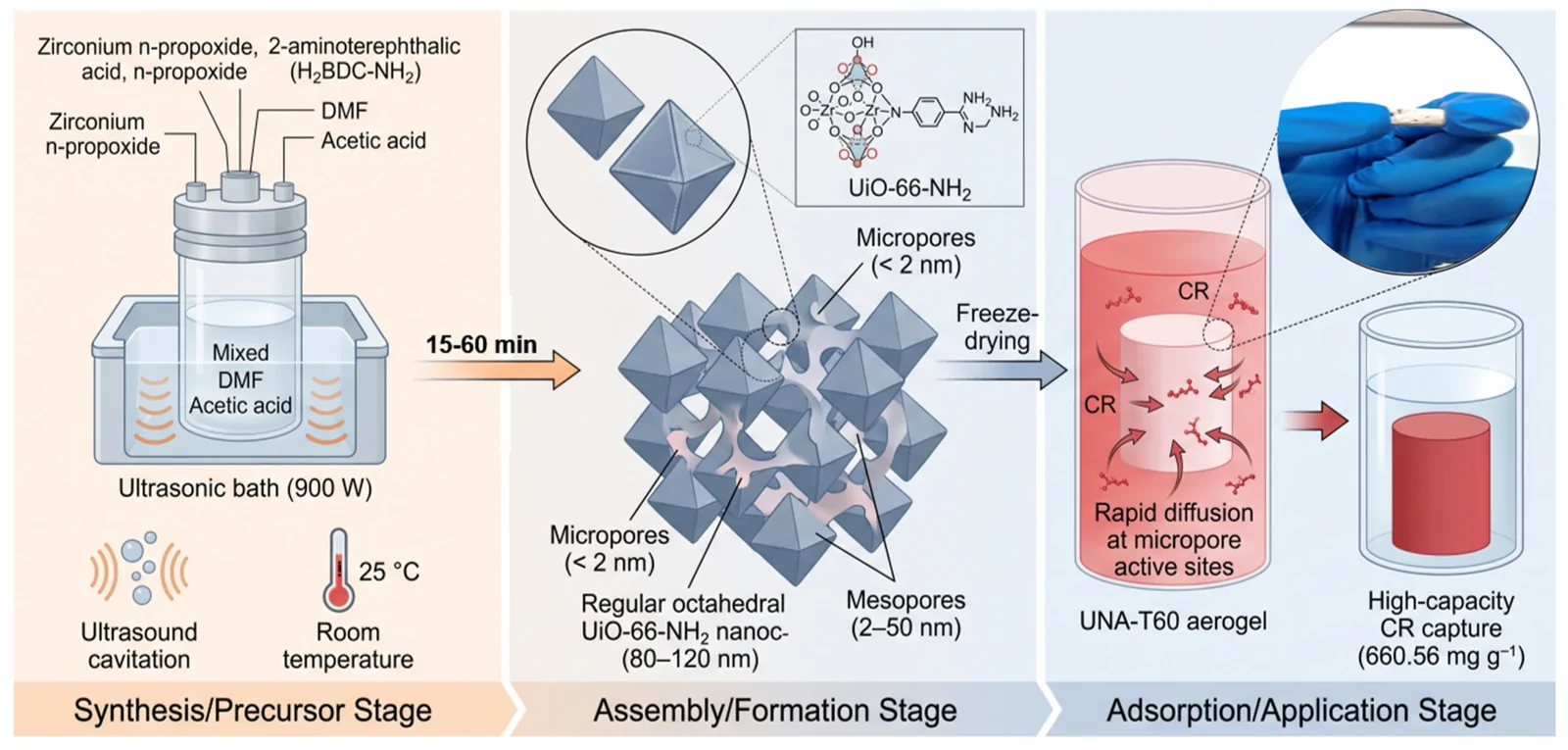
Schematic illustration of the room-temperature ultrasound-assisted synthesis and adsorption application of micro-/mesoporous UiO-66-NH_2_ self-assembled aerogels (UNA-Tt). (**Left**) Precursor mixture undergoing ultrasonic cavitation at 900 W and 25 °C. (**Middle**) In situ self-assembly of regular octahedral UiO-66-NH_2_ nanocrystals (80–120 nm) into a three-dimensional aerogel framework with intrinsic micropores (<2 nm) and intercrystalline mesopores (2–50 nm) via freeze-drying. (**Right**) High-capacity CR capture (660.56 mg g^−1^) by UNA-T60 aerogel through rapid diffusion into hierarchical pore channels.

The exceptional cyclic stability of UNA-T60, which retains 90.8% of its initial adsorption capacity after four regeneration cycles, is attributed to its regular octahedral framework structure and highly crystalline, as directly evidenced by well-defined crystal morphology observed by SEM and the sharp XRD peaks. The robust intercrystalline contacts within the self-assembled aerogel effectively resist structural collapse during repeated ultrasonic regeneration, thereby preserving the bimodal micro-/mesoporous architecture. In this architecture, intrinsic micropores continue to provide high-density adsorption sites while intercrystalline mesopores maintain efficient molecular transport pathways, ensuring that the adsorption performance is retained across multiple cycles. In contrast, the markedly inferior stability of UNA-T15 (75.2% retention) correlates with its low-crystallinity, amorphous aggregate morphology (Figure S1), which lacks the mechanical integrity to withstand repeated ultrasonic treatment and is prone to progressive structural disintegration. Consequently, crystallinity and structural integrity—both directly governed by sonication time—are identified as the decisive factors controlling reusability. The overall ultrasound-assisted synthesis strategy and the resulting bimodal micro-/mesoporous network responsible for the high-capacity CR capture and sustained regeneration performance are conceptually summarized in Scheme 1.

## 2. Results and Discussion

In this study, the micro-/mesoporous UiO-66-NH_2_ self-assembled aerogels synthesized via room-temperature ultrasound-assisted direct synthesis are designated as UNA-Tt, where U denotes Ultrasound, N denotes ambient (room-temperature), A denotes Aerogel, and Tt represents the sonication time (min). Accordingly, samples prepared at 15, 30, 45, and 60 min are denoted as UNA-T15, UNA-T30, UNA-T45, and UNA-T60, respectively.

**Figure 1:**
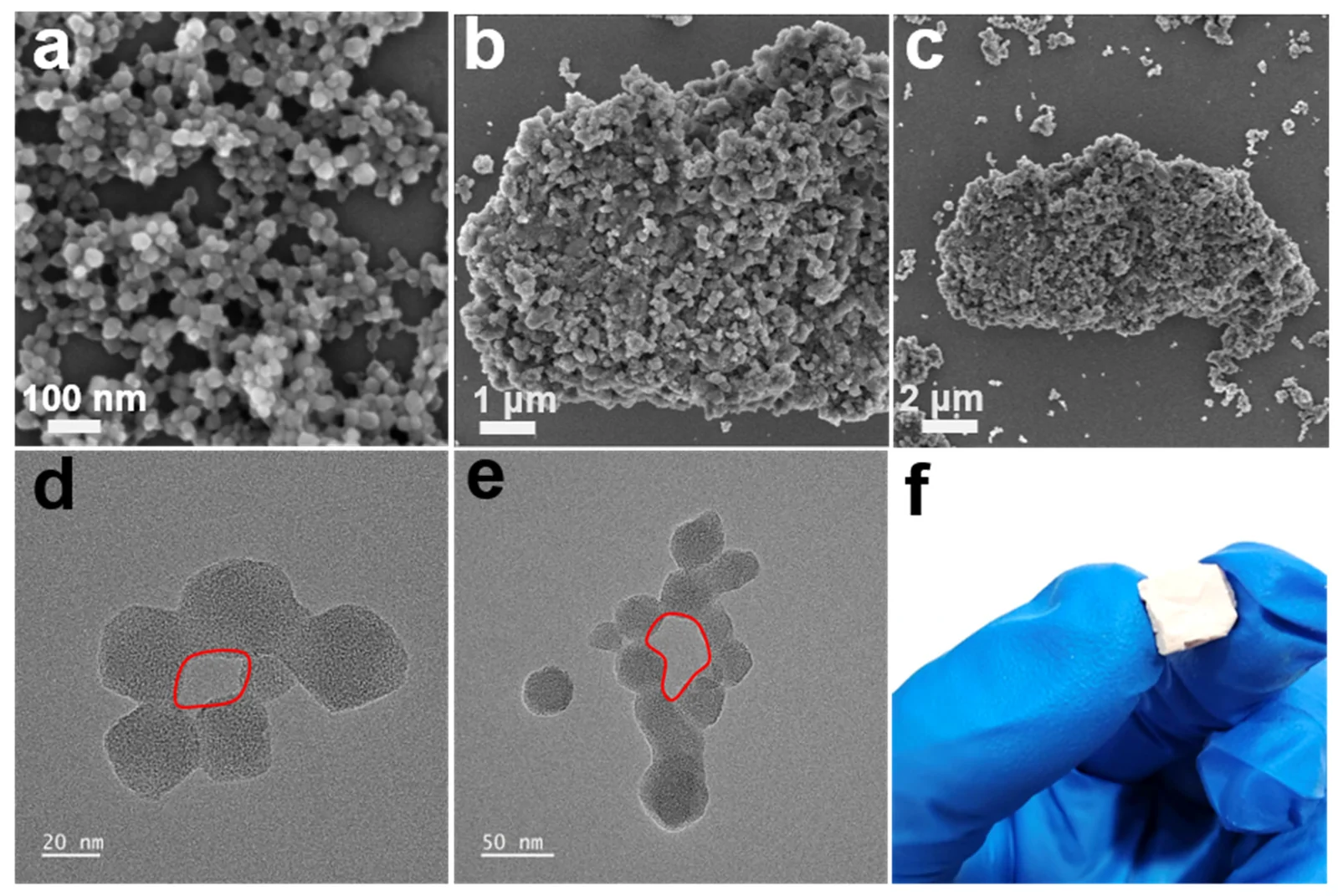
Morphological characterization of UNA-T60. (**a**–**c**) Scanning electron microscopy (SEM) images at various magnifications. (**d**,**e**) Transmission electron microscopy (TEM) images. (**f**) Digital photograph of the as-prepared material.

The microstructural morphology and macroscopic shaping characteristics of UNA-T60 were systematically characterized by scanning electron microscopy (SEM) and transmission electron microscopy (TEM), with the results presented in Figure 1.

**Figure 2:**
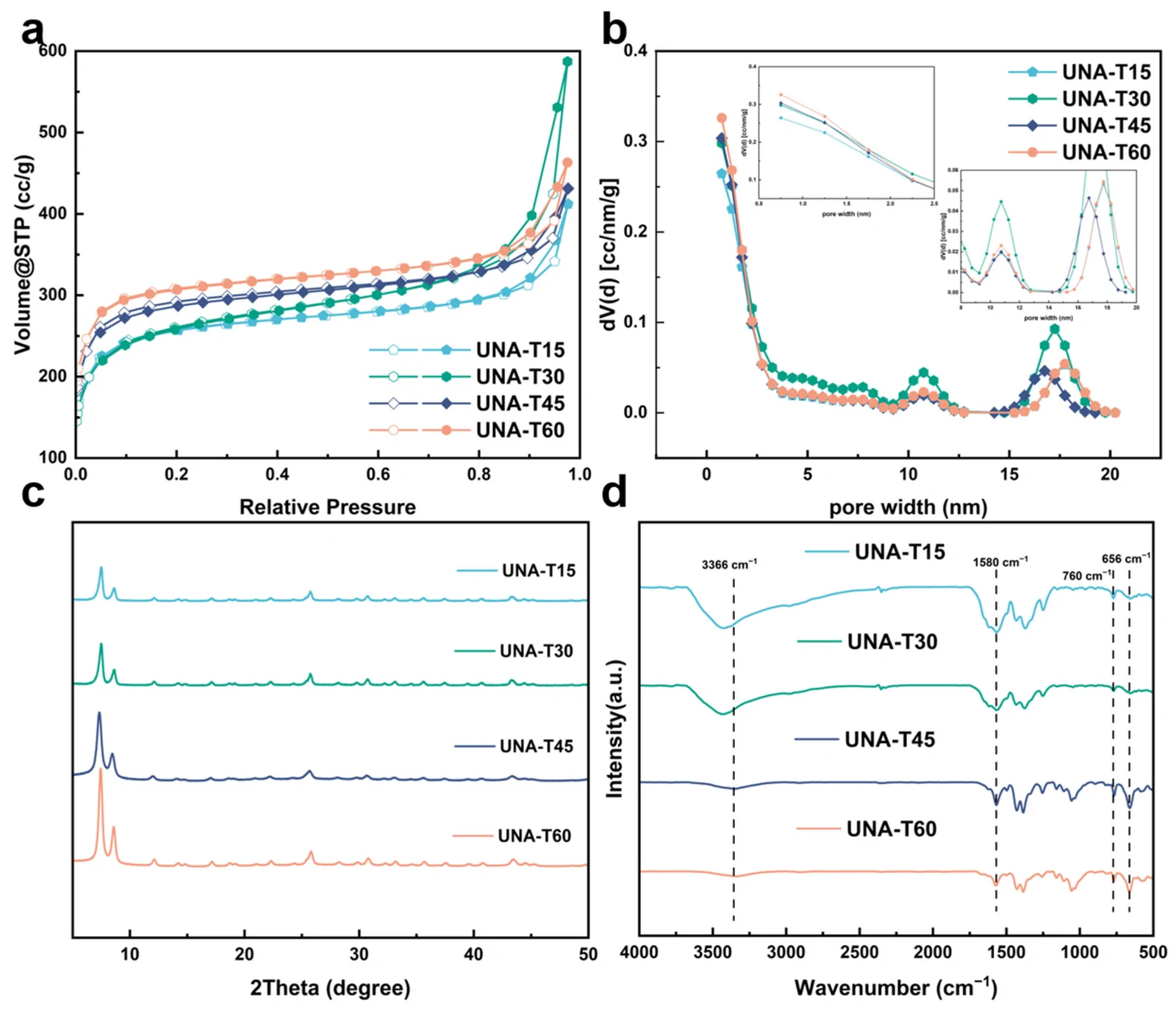
(**a**) Nitrogen adsorption–desorption isotherms, (**b**) pore size distribution curves, (**c**) X-ray diffraction (XRD) patterns, and (**d**) Fourier-transform infrared (FTIR) spectra of the UNA-Tt series.

Figure 1a presents a high-magnification SEM image of UNA-T60 (scale bar 100 nm), clearly revealing densely packed, highly uniform regular octahedral nanocrystals with well-developed crystal facets and sharp edges, exhibiting particle sizes of approximately 80–120 nm. The nanocrystals are mutually supported via point or face contacts, generating abundant intercrystalline pores that constitute a three-dimensional interconnected network. Figure 1b shows a medium-magnification SEM image (scale bar 1 μm), in which the nanocrystal assemblies exhibit a loosely porous, sponge-like architecture with high surface roughness and uniform pore distribution. Figure 1c displays a low-magnification SEM image (scale bar 2 μm), demonstrating that UNA-T60 is composed of several micron-sized aggregates loosely interconnected with one another; the macroscopic voids between aggregates are qualitatively visible and appear to interconnect with the mesoscopic pores between crystallites. It should be noted that these inter-aggregate voids fall within the macropore regime (>50 nm), which is beyond the reliable quantification range of the N_2_ adsorption–desorption analysis employed in this study (see Figure 2 and Table 1. Figure 1d,e present TEM images of UNA-T60. Figure 1d (scale bar 20 nm) shows the high-resolution morphology of an individual regular octahedral nanocrystal, with clearly discernible lattice fringes; the region highlighted by the red box indicates pores formed by interparticle packing, confirming its excellent crystallinity. Figure 1e (scale bar 50 nm) illustrates the aggregation state of multiple nanocrystals, with distinct mesoporous gaps evident between crystallites; the region highlighted by the red box similarly demonstrates pores generated by interparticle packing, consistent with the disordered intercrystalline packing features observed by SEM and further corroborating the formation mechanism of mesoporous channels. Figure 1f presents a digital photograph of UNA-T60, showing a pinkish-white monolithic material with a lightweight texture that can be readily handled with tweezers, exhibiting good mechanical integrity and self-supporting properties. This macroscopic shaping characteristic overcomes the inherent limitation of conventional MOF powders regarding difficult separation and recovery, providing operational convenience for engineering applications such as fixed-bed adsorption. The aforementioned morphological characterization results indicate that 60 min of ambient-temperature ultrasonic treatment promotes the full growth and spontaneous assembly of UiO-66-NH_2_ nanocrystals, yielding a self-supporting monolithic aerogel material with an interconnected bimodal micro-/mesoporous network. It should be noted that, in the absence of a static control experiment prepared under identical conditions without ultrasound, the specific causal role of ultrasound in driving this crystallization and assembly process cannot be unequivocally established from the present data alone. The complete development of regular octahedral nanocrystals provides abundant intrinsic microporous adsorption sites, while the mesoporous network formed by intercrystalline packing serves as molecular transport channels that significantly reduce mass transfer resistance. This synergistic “micropore–mesopore” bimodal architecture constitutes the structural foundation for the superior adsorption performance of UNA-T60.

The SEM images of UNA-T15, UNA-T30, and UNA-T45 are presented in Figure S1 of the Supplementary Materials. UNA-T15 exhibits an amorphous aggregated morphology with no discernible crystal facet development. UNA-T30 displays increased nanocrystallite dimensions with still indistinct contours, with incipient mesoporous channels becoming apparent. UNA-T45 shows partially well-developed polyhedral nanocrystals coexisting with an amorphous matrix. The aforementioned morphological evolution sequence, in contrast with the intact regular octahedral structure of UNA-T60, collectively elucidates the decisive regulatory role of sonication time in governing the nucleation, growth, and self-assembly processes of UiO-66-NH_2_ nanocrystals.

The pore structural characteristics of the UNA-Tt series samples were characterized by nitrogen adsorption–desorption measurements at 77 K, with the results presented in Figure 2 and Table 1. Figure 2a displays the nitrogen adsorption–desorption isotherms for the four samples. All samples exhibit typical Type IV isotherm characteristics, with pronounced H3-type hysteresis loops observed in the relative pressure (P/P_0_) range of 0.4–0.9, indicating the presence of slit-like mesoporous structures formed by the disordered packing of nanocrystals. Additionally, the sharp uptake in the low relative pressure region (P/P_0_ < 0.1) reflects micropore filling behavior, confirming the existence of intrinsic micropores of UiO-66-NH_2_.

The specific surface area and pore structure parameters are summarized in Table 1. The BET specific surface areas (S_BET_) of UNA-T15, UNA-T30, UNA-T45, and UNA-T60 are 950.7, 957.7, 1127.6, and 1196.9 m^2^ g^−1^, respectively; the corresponding Langmuir specific surface areas are 1435.7, 1739.5, 1517.7, and 1656.8 m^2^ g^−1^; and the total pore volumes are 0.6375, 0.9082, 0.6672, and 0.7166 cm^3^ g^−1^, respectively. Overall, the BET specific surface area exhibits an increasing trend with prolonged sonication time. UNA-T15 and UNA-T30 show comparable values (approximately 950 m^2^ g^−1^), whereas UNA-T45 and UNA-T60 demonstrate significant enhancement (>1100 m^2^ g^−1^), indicating that the refinement of crystal structure beyond 45 min of sonication exerts a decisive promoting effect on pore development. Notably, UNA-T30 exhibits the highest Langmuir specific surface area (1739.5 m^2^ g^−1^) and total pore volume (0.9082 cm^3^ g^−1^) among all samples, which may be attributed to its highest mesopore proportion (71.5%). The analysis of micropore and mesopore proportions reveals the delicate regulatory effect of sonication time on pore structure evolution (Table 1). UNA-T15 possesses comparable micropore and mesopore proportions of 52.1% and 47.9%, respectively. For UNA-T30, the mesopore proportion increases sharply to 71.5% while the micropore proportion decreases to 28.5%, suggesting a lower degree of nanocrystal assembly under this condition, with substantial mesoporosity generated by intercrystalline packing. For UNA-T45 and UNA-T60, the micropore proportions recover to 58.7% and 57.2%, respectively, with corresponding mesopore proportions decreasing to 41.3% and 42.8%. This indicates that extended sonication time promotes the refinement of crystal structure and the development of intrinsic micropores, while the improved size uniformity of nanocrystals enables more compact intercrystalline packing, leading to a stabilized mesopore proportion.

Figure 2b presents the pore size distribution curves calculated based on the density functional theory (DFT) model. All samples exhibit two distinct micropore peaks at approximately 0.6–0.8 nm and 1.0–1.2 nm, corresponding to the tetrahedral cage and octahedral cage apertures of UiO-66-NH_2_, respectively. Concurrently, a broad mesopore distribution peak appears in the 10–20 nm range, originating from the interstitial packing gaps between nanocrystals. The inset magnification of the micropore region (0.5–2.5 nm) reveals that UNA-T45 and UNA-T60 display significantly higher micropore peak intensities than UNA-T15 and UNA-T30, consistent with their higher micropore proportions listed in Table 1. The magnified view of the mesopore region (8–20 nm) shows that UNA-T30 possesses the highest mesopore peak intensity and the broadest distribution, further corroborating its structural characteristic of the largest mesopore proportion. These findings are in good agreement with the SEM observations.

The above results indicate that ultrasonic energy input induces significant changes in the micro-/mesoporous architecture of the resulting UiO-66-NH_2_ aerogels. It should be emphasized that the BET and DFT analyses employed here reliably characterize the micropore (<2 nm) and mesopore (2–50 nm) regimes; however, they are inherently insensitive to macropores (>50 nm) due to the absence of capillary condensation in this range. While low-magnification SEM (Figure 1c) qualitatively reveals inter-aggregate voids that may fall within the macropore regime, their size distribution, pore volume, and quantitative contribution to adsorption were not determined in this study. Under a shorter sonication time (15 min), crystal growth is incomplete, with comparable proportions of micropores and mesopores but a limited overall specific surface area. Moderately extending the sonication time (30 min) facilitates the formation of abundant mesoporous channels (mesopore proportion of 71.5%), yet insufficient micropore development prevents a marked increase in the BET specific surface area. When the sonication time reaches 45–60 min, the nanocrystals develop into complete regular octahedra, the micropore proportion recovers to approximately 58%, while an appropriate amount of mesopores is retained as mass-transfer channels, achieving synergistic optimisation of the bimodal micro-/mesoporous hierarchy. The specific surface area of UNA-T60 (1196.9 m^2^ g^−1^) approaches the typical level of UiO-66-NH_2_ powders synthesised by solvothermal methods (1200–1400 m^2^ g^−1^), demonstrating that the room-temperature ultrasound-assisted strategy preserves a high specific surface area associated with the well-developed micropore–mesopore hierarchy, while simultaneously achieving macroscopic shaping of the material.

The crystal structures of the UNA-Tt series samples were characterised by X-ray diffraction (XRD), with the results presented in Figure 2c. All samples exhibited sharp diffraction peaks at 2θ = 7.3°, 8.5°, 12.1°, 14.2°, 17.5°, 25.8°, and 33.2°, which are consistent with the simulated standard diffraction peak positions of UiO-66-NH_2_, corresponding to the (111), (200), (222), (400), (511), (731), and (751) crystal planes of UiO-66-NH_2_, respectively. This indicates that the samples prepared under different sonication times all possess a pure-phase UiO-66-NH_2_ crystal structure, with no diffraction peaks of ZrO_2_ or other zirconium-based impurity phases detected, confirming that the room-temperature ultrasound-assisted strategy successfully achieves the controlled synthesis of UiO-66-NH_2_.

Further comparison of the diffraction peak characteristics of each sample reveals that with increasing sonication time, the diffraction peak intensities gradually increase, and the peak shapes become progressively sharper. Specifically, UNA-T15 exhibits the lowest diffraction peak intensities with broadened peak profiles, indicating a lower degree of crystallinity, smaller crystal sizes, or a higher density of lattice defects, which is consistent with the amorphous aggregate morphology observed by SEM. UNA-T30 shows a slight increase in diffraction peak intensity, yet the (111) peak remains significantly broadened, suggesting incomplete crystal growth. UNA-T45 exhibits markedly sharper diffraction peaks with improved resolution of individual crystal plane peaks, indicating a notable enhancement in crystallinity. UNA-T60 displays the sharpest diffraction peaks with the highest intensities, demonstrating the most developed crystal structure, the largest crystallite sizes, and the highest crystallinity, which is in excellent agreement with the abundant well-defined regular octahedral nanocrystal morphology observed in SEM.

The aforementioned crystallinity evolution pattern indicates that the energy input provided by the ultrasonic cavitation effect effectively promotes crystal nucleation and growth, enabling the nanocrystals to progressively develop from disordered low-crystallinity intermediates into highly crystalline regular octahedral crystals. This process of crystal structure perfection corroborates the recovery trend of micropore proportion observed in the preceding BET analysis (UNA-T30→ UNA-T45→ UNA-T60): at the UNA-T30 stage, the mesopore proportion is the highest (71.5%) while the micropore proportion is the lowest (28.5%), corresponding to the broadened diffraction peaks and insufficient crystallinity in XRD; at the UNA-T45 and UNA-T60 stages, the micropore proportion recovers to approximately 58%, corresponding to the significantly sharpened diffraction peaks and enhanced crystallinity in XRD. The cross-validation of multiple characterisation techniques collectively reveals the decisive regulatory role of sonication time on the structural evolution of UiO-66-NH_2_ aerogels—sufficient ultrasonic energy input is the key to driving the synergistic optimisation of crystal perfection and micro-/mesoporous network.

The surface functional groups and chemical bond vibrations of the UNA-Tt series samples were characterised by Fourier-transform infrared (FTIR) spectroscopy, with the results shown in Figure 2d. All samples exhibited spectral bands that are highly consistent with the standard characteristic absorption peaks of UiO-66-NH_2_, confirming that the target MOF structure was successfully constructed under different sonication times. In the low-wavenumber region (500–800 cm^−1^), the absorption bands at 656 cm^−1^ and 760 cm^−1^ are assigned to the symmetric stretching vibration and bending vibration modes of the Zr–O bond, respectively, confirming that the secondary building unit (SBU) composed of zirconium oxo clusters (Zr_6_O_4_(OH)_4_^12+^) was well preserved during the ultrasonic synthesis process. The absorption bands in the range of 1400–1500 cm^−1^ are attributed to the symmetric stretching vibration of the carboxylate groups (–COO-), the strong absorption signal near 1580 cm^−1^ corresponds to the coupled peak of the aromatic ring skeleton (C=C) stretching vibration and the asymmetric stretching vibration of carboxylate groups, and the absorption bands in the 1250–1350 cm^−1^ range correspond to the C–N stretching vibration. The coexistence of these characteristic peaks indicates that the organic ligand H2BDC–NH_2_ has been stably integrated into the metal–organic framework through coordination bonds, forming a complete Zr–O–C coordination structure, and that the amino functional groups were successfully introduced into the framework. In the 3300–3500 cm^−1^ region, all samples exhibit an absorption peak near 3366 cm^−1^, which is attributed to the N–H stretching vibration of the –NH_2_ functional groups. Notably, the peak shape of this absorption band exhibits a regular evolution with increasing sonication time: the N–H peak of UNA-T15 shows the most significant broadening, with a full width at half maximum (FWHM) considerably larger than that of the other samples; UNA-T30 still exhibits a relatively broad peak shape; UNA-T45 and UNA-T60 show a reduced degree of broadening. This progressive sharpening is ascribed to the regulatory effect of sonication time on the surface structural order of the material. Under shorter sonication time (UNA-T15), the nanocrystals are incompletely developed, with numerous amorphous fusion regions on the surface (see Supplementary Materials, Figure S1a), and the –NH_2_ groups are situated in highly diverse local chemical environments, leading to significant broadening of the N–H vibration peak. As the sonication time increases, the crystal structure becomes more perfect (UNA-T45, UNA-T60), the surface regularity improves, and the chemical environment homogeneity of the –NH_2_ groups is enhanced, resulting in a corresponding decrease in the degree of N–H peak broadening. However, even when the sonication time is extended to 60 min, due to the persistence of intercrystalline fusion regions between nanocrystals (see Supplementary Materials, Figure S1j), some –NH_2_ groups remain in a non-uniform chemical environment, and thus the N–H peak fails to become completely sharpened. This spectral evolution trend is highly consistent with the SEM observations of the crystal surface morphology transitioning from disordered fusion to regular octahedra while retaining local fusion structures, further confirming the unique role of the ultrasonic synthesis strategy in regulating the surface chemical state of the material.

**Figure 3:**
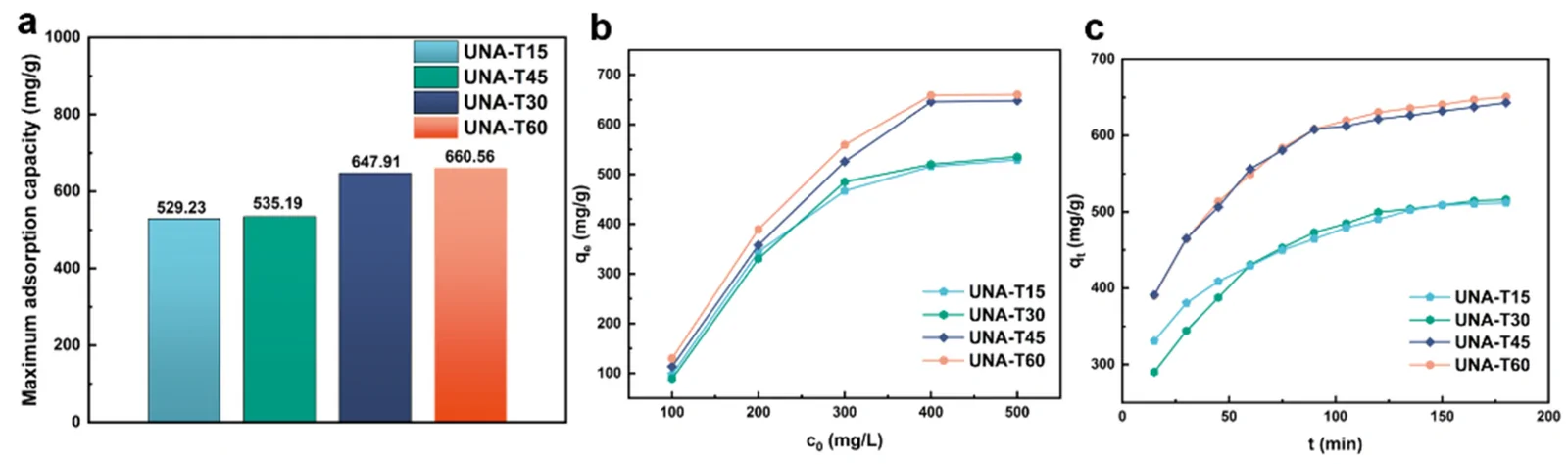
UNA-Tt for CR: (**a**) maximum adsorption capacity, (**b**) adsorption capacity at different concentrations, and (**c**) time-dependent adsorption curve at 400 mg L^−1^.

The adsorption performance of the UNA-Tt series samples toward CR was investigated by static adsorption experiments. Figure 3a presents a comparison of the maximum adsorption capacities of each sample at room temperature. The maximum adsorption capacities (q_m_) of UNA-T15, UNA-T30, UNA-T45, and UNA-T60 are 529.23, 535.19, 647.91, and 660.56 mg g^−1^, respectively. With increasing sonication time, the adsorption capacity exhibits an increasing trend, among which UNA-T45 and UNA-T60 show a significant enhancement compared to UNA-T15/30 (an increase of approximately 22.4–23.7%), indicating that sonication treatment exceeding 45 min is the key condition for obtaining UiO-66-NH_2_ aerogels with high adsorption capacity.

Figure 3b presents the isothermal adsorption curves of the equilibrium adsorption capacity (q_e_) of each sample as a function of the initial concentration (c_0_). In the range of c_0_ = 100–500 mg L^−1^, the q_e_ values of all samples increase with increasing c_0_, but the growth rate gradually slows down, exhibiting a typical saturation adsorption trend. Specifically, UNA-T15 and UNA-T30 show rapid q_e_ growth in the low-concentration region (c_0_ < 200 mg L^−1^) but tend to level off in the high-concentration region (c_0_ > 300 mg L^−1^), eventually stabilising at approximately 515.92 and 520.24 mg g^−1^, respectively. In contrast, UNA-T45 and UNA-T60 maintain a relatively high growth rate over the entire concentration range, with q_e_ reaching 645.80 and 658.96 mg g^−1^ at c_0_ = 400 mg L^−1^, respectively, approaching their maximum adsorption capacities, indicating that both materials possess stronger affinity toward CR and higher utilisation efficiency of active sites.

Figure 3c shows the kinetic curves of the adsorption capacity (q_t_) of each sample as a function of time at c_0_ = 400 mg L^−1^. All samples exhibit rapid adsorption characteristics: in the initial stage (0–30 min), q_t_ increases sharply, with the initial adsorption rates of UNA-T60 and UNA-T45 being significantly higher than those of UNA-T15 and UNA-T30; subsequently, the adsorption enters a slow growth stage (30–120 min), during which the adsorption rate gradually decreases; finally, adsorption equilibrium is reached within 120–180 min. The equilibrium adsorption capacities (q_e,Exp_) of UNA-T60, UNA-T45, UNA-T30, and UNA-T15 at 180 min are 650.57, 642.97, 516.25, and 511.94 mg g^−1^, respectively, which are consistent with the isotherm test results.

The correlation between pore architecture and adsorption capacity is further elucidated by comparing the structural parameters across the UNA-Tt series (Table 1). Notably, UNA-T30 exhibits the highest total pore volume (0.9082 cm^3^ g^−1^) and the largest mesopore proportion (71.5%), yet its maximum adsorption capacity (535.19 mg g^−1^) remains comparable to that of UNA-T15 and significantly lower than those of UNA-T45 and UNA-T60. This observation demonstrates that mesoporosity alone is insufficient to ensure high adsorption capacity. In contrast, UNA-T45 and UNA-T60, which possess recovered micropore proportions of ~58% and markedly enhanced BET specific surface areas (>1100 m^2^ g^−1^), achieve substantially higher capacities (647.91 and 660.56 mg g^−1^, respectively). These data establish a clear structure–performance relationship: the synergistic coexistence of intrinsic micropores (providing high-density adsorption sites) and intercrystalline mesopores (providing low-resistance diffusion pathways) is the primary structural origin of the superior CR capture performance, rather than any single pore regime or unverified surface chemical mechanism.

**Figure 4:**
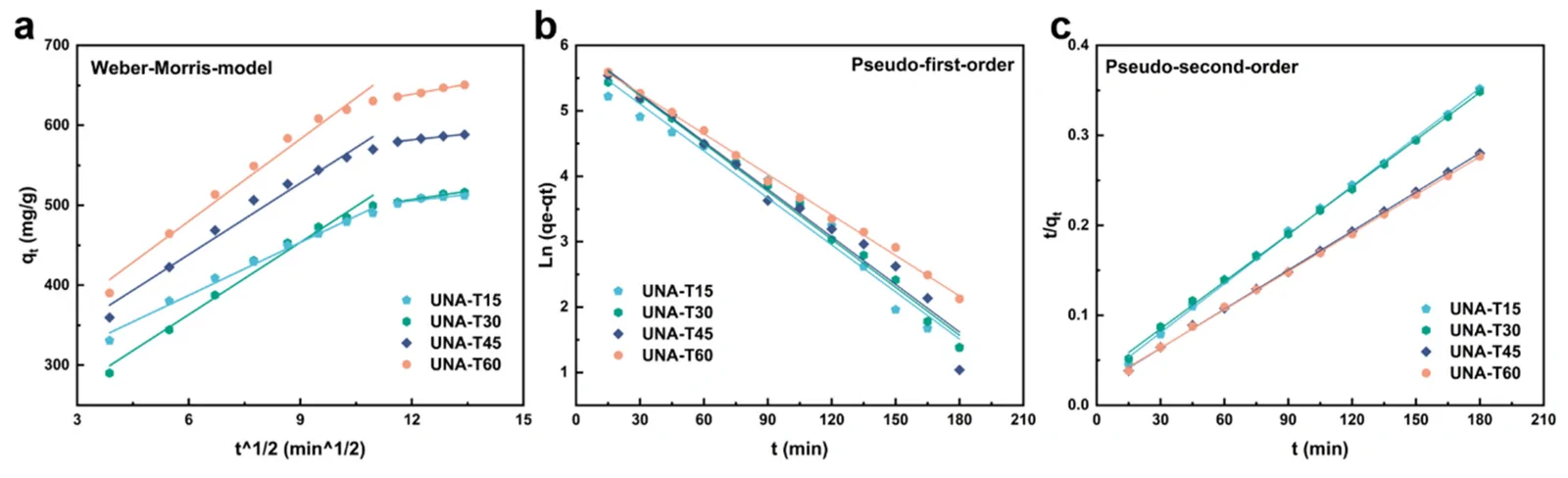
UNA-Tt for CR: (**a**) Weber–Morris’s intraparticle diffusion model, (**b**) pseudo-first-order kinetic plots, and (**c**) pseudo-second-order kinetic plots.

To gain deeper insight into the adsorption mechanism, the kinetic data were fitted using the pseudo-first-order kinetic model, the pseudo-second-order kinetic model, and the Weber–Morris intraparticle diffusion model, with the results shown in Figure 4 and the fitting parameters listed in Table S1 of the Supplementary Materials. Figure 4a presents the fitting curves of the Weber–Morris intraparticle diffusion model (q_t_ vs. t^0.5^). The q_t_ vs. t^0.5^ plots for all samples exhibit two distinct linear segments, indicating that the adsorption process comprises two rate-controlling stages. The first segment (t^0.5^ ≈ 3.9–11.6 min^0.5^, corresponding to t ≈ 15–135 min) exhibits a larger slope, with intraparticle diffusion rate constants (K_p1_) of 22.04, 30.90, 33.20, and 34.27 mg·g^−1^·min^−0.5^, respectively, corresponding to the rapid diffusion of CR molecules from the bulk solution to the external surface of the adsorbent and their subsequent occupation of surface active sites. The second segment (t^0.5^ ≈ 11.6–13.4 min^0.5^, corresponding to t ≈ 135–180 min) shows a significantly decreased slope, with K_p2_ values of 5.22, 7.08, 9.15, and 8.52 mg g^−1^ min^−0.5^, respectively, corresponding to the slow diffusion of CR molecules within the mesoporous channels and the gradual occupation of micropore active sites. The intercepts (I_1_ and I_2_) of both linear segments are non-zero, indicating that intraparticle diffusion is one of the rate-controlling steps of the adsorption process, but not the sole rate-limiting factor; boundary layer diffusion and external surface adsorption processes also contribute significantly to the overall adsorption rate. The higher K_p1_ and K_p2_ values of UNA-T45 and UNA-T60 compared to those of UNA-T15 and UNA-T30 indicate that their more well-developed micro-/mesoporous network effectively promotes the rapid mass transfer and deep diffusion of CR molecules.

A detailed comparison of UNA-T60 with recently reported MOF aerogels and UiO-66-NH_2_-based adsorbents, covering synthesis conditions, specific surface areas, adsorption capacities, and regeneration performance, is provided in Supplementary Materials’ Table S2.

Figure 4b presents the linear fitting plots of the pseudo-first-order kinetic model (ln(q_e_ − q_t_) vs. t). The fitting curves of all samples show a certain linear trend; however, the data points in the high-time region (t > 120 min) deviate significantly from the straight line, indicating that the pseudo-first-order kinetic model fails to adequately describe the adsorption process of CR onto UNA-Tt. The corresponding fitting parameters (see Table S1 in the Supplementary Materials) show that the calculated equilibrium adsorption capacities (q_e,Cal_) from the pseudo-first-order model for UNA-T15, UNA-T30, UNA-T45, and UNA-T60 are 338.30, 389.98, 396.49, and 359.67 mg g^−1^, respectively, which deviate considerably from the experimental equilibrium adsorption capacities (q_e,Exp_ = 511.94, 516.25, 642.97, and 650.57 mg g^−1^). Although the correlation coefficients (R^2^) are 0.97296, 0.99186, 0.97115, and 0.9970917, respectively, which are relatively high, the inconsistency between q_e,Cal_ and q_e,Exp_ indicates the limited applicability of this model, which is consistent with the literature reports that the pseudo-first-order kinetic model is generally applicable only to the initial stage of adsorption (the first 20–30 min).

To enable a rigorous quantitative comparison beyond visual inspection of linearity, the predictive accuracy of each model was further assessed by the relative error between calculated and experimental equilibrium capacities (|q_e,Cal_ − q_e,Exp_|/q_e,Exp_ × 100%). For the pseudo-first-order model, the relative errors are 33.9%, 24.5%, 38.3%, and 44.7% for UNA-T15, UNA-T30, UNA-T45, and UNA-T60, respectively, indicating substantial systematic underestimation of the equilibrium capacity. In contrast, the pseudo-second-order model yields relative errors of only 7.9%, 10.7%, 7.3%, and 8.2%, respectively. The normalized standard errors of the estimate (SNE) further confirm the superior performance of the pseudo-second-order model (SNE < 0.05) compared with the pseudo-first-order model (SNE > 0.15). Nevertheless, we recognize that linearization of kinetic models can distort error structures and artificially inflate correlation coefficients. To circumvent this limitation, both models were additionally fitted to the untransformed experimental data (q_t_ versus t) using nonlinear least-squares regression. The PFO model in its nonlinear form (q_t_ = q_e_ [1 − Exp(−k_1_t)]) yields R^2^ values of only 0.744–0.884, with RMSE values ranging from 24.14 to 29.50 mg g^−1^ (Table S1). In contrast, the nonlinear PSO model (q_t_ = k_2_q_e_^2^t/(1 + k_2_q_e_t)) achieves markedly higher R^2^ values (0.940–0.978) and substantially lower RMSE (11.30–13.61 mg g^−1^). Furthermore, the Akaike information criterion (AIC) and Bayesian information criterion (BIC) consistently favor the PSO model over PFO for all samples (Table S1). Notably, the nonlinear PFO curves systematically underestimate qt at longer contact times (t > 90 min), corroborating the deviation observed in the linear plots but now quantified on the original scale (Figure S2). These results demonstrate that the high linear R^2^ values previously reported for the PFO model were indeed an artifact of the linearization procedure. Collectively, the nonlinear regression analysis provides robust statistical evidence that the adsorption of CR onto UNA-Tt is best described by the pseudo-second-order model.

Figure 4c presents the linear fitting plots of the pseudo-second-order kinetic model (t/q_t_ vs. t). The fitting curves of all samples exhibit excellent linear correlations (R^2^ > 0.998), with data points closely distributed on both sides of the straight lines. Crucially, the pseudo-second-order model not only yields higher R^2^ values than the pseudo-first-order model but also provides calculated equilibrium capacities (q_e,Cal_) in excellent agreement with the experimental values (relative error < 11%), as quantified above. The calculated equilibrium adsorption capacities (q_e,Cal_) from the pseudo-second-order model are 552.49, 571.43, 689.66, and 704.23 mg g^−1^, respectively, which are in excellent agreement with the experimental q_e,Exp_ values, and the R^2^ values are all higher than 0.998 (UNA-T15: 0.99845; UNA-T30: 0.99907; UNA-T45: 0.99950; UNA-T60: 0.99944). The above results indicate that the adsorption process of CR onto UNA-Tt is well described by the pseudo-second-order model (R^2^ > 0.998). It is critically noted that the PSO model describes the overall rate-limiting step as surface adsorption-controlled, but it does not discriminate between physical adsorption and chemical adsorption, nor does it identify specific intermolecular forces. The excellent PSO fit, combined with the high intraparticle diffusion rate constants (K_p1_ and K _p2_) observed for UNA-T45 and UNA-T60 (Table S1), indicates that the hierarchical pore architecture plays the decisive role in governing adsorption kinetics: mesopores (2–50 nm) function as molecular transport corridors that minimize mass-transfer resistance, while micropores (<2 nm) provide the abundant surface area required for rapid site saturation. Therefore, the observed kinetic behavior is interpreted as a structure-enabled, surface adsorption-limited process rather than evidence for any specific chemical bonding mechanism.

To reveal the adsorption thermodynamic behaviour of the UNA-Tt series samples toward CR, the equilibrium adsorption data at room temperature were fitted using three isotherm models, namely Langmuir, Freundlich, and Temkin. Prior to model fitting, the data quality at each concentration point was systematically evaluated. Initially, all five concentration points (100–500 mg L^−1^) were included in the linear regression analysis of the Langmuir model (c_e_/q_e_ vs. c_e_). It was found that the 100 mg L^−1^ data point exhibited a significant deviation: its c_e_/q_e_ value was considerably higher than the linear trend displayed by the other data points, resulting in extremely low R^2^ values (0.001–0.031) for the five-point fitting, and the calculated theoretical maximum adsorption capacity (q_m,Cal_) yielded negative values or absurdly large values (>10,000 mg g^−1^), which are physically unreasonable. This phenomenon arises from a non-equilibrium effect under low initial concentration conditions. At c_0_ = 100 mg L^−1^, the concentration gradient-and thus the mass transfer driving force is insufficient to overcome the boundary layer resistance within the contact time of 180 min. Consequently, the measured q_e_ values (89.12–130.08 mg g^−1^) underestimate the true equilibrium adsorption capacities, rendering this point a statistical outlier. It is critical to note that the Langmuir and Freundlich models are derived from the fundamental premise that the system has attained thermodynamic equilibrium. Inclusion of a non-equilibrium data point violates this core assumption and produces physically meaningless parameters (negative or >10,000 mg g^−1^ q_m,Cal_), rather than merely lowering the correlation coefficient. Therefore, exclusion of the 100 mg L^−1^ point is not a curve-fitting convenience but a necessary step to uphold the physical validity of the model. Linear regression was subsequently performed using the four equilibrium points (200–500 mg L^−1^). This approach of omitting non-equilibrium outliers from isotherm fitting is well documented in the adsorption literature.

This phenomenon can be attributed to the non-equilibrium effect under low initial concentration conditions: at c_0_ = 100 mg L^−1^, the mass transfer driving force between the adsorbate and the adsorbent is relatively small, and true thermodynamic equilibrium may not have been reached within the contact time of 180 min. Under low-concentration conditions, CR molecules require a longer time to overcome the boundary layer resistance and fully occupy the active sites on the adsorbent surface; therefore, the measured q_e_ values (89.12–130.08 mg g^−1^) are likely lower than the true equilibrium adsorption capacities, causing this data point to behave as a statistical outlier in the isotherm model fitting. Based on the above analysis, to ensure the physical rationality and statistical reliability of the isotherm model parameters, the 100 mg L^−1^ data point was excluded from the fitting of the Langmuir and Freundlich models, and linear regression analysis was performed using only the four concentration points of 200, 300, 400, and 500 mg L^−1^. This data processing approach has precedents in adsorption studies.

**Figure 5:**
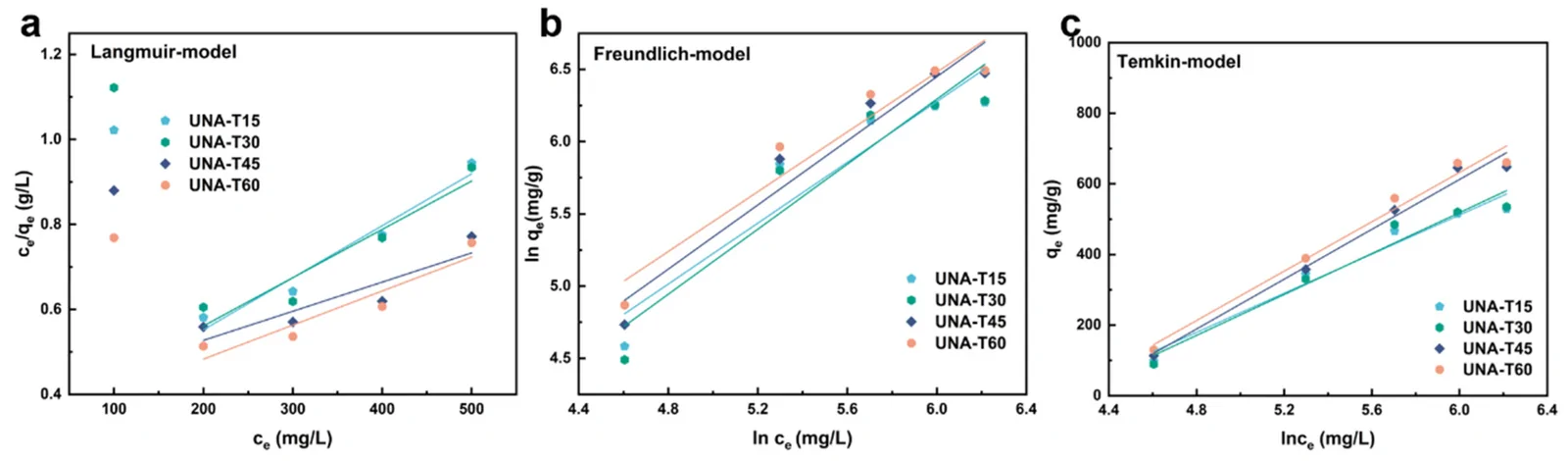
UNA-Tt for CR: (**a**) Langmuir’s linear fitting plot (excluding 100 mg L^−1^), (**b**) Freundlich’s linear fitting plot (excluding 100 mg L^−1^), and (**c**) Temkin’s linear fitting plot.

Figure 5a presents the linear fitting plots of the Langmuir model (c_e_/q_e_ vs. c_e_). The fitting curves of all samples exhibit good linear correlations; however, the data points of UNA-T45 and UNA-T60 in the high-concentration region (c_e_ > 400 mg L^−1^) deviate slightly from the straight line, indicating that the assumption of homogeneous monolayer adsorption in the Langmuir model exhibits some deviation at extremely high concentrations. The theoretical maximum adsorption capacities (q_m,Cal_) obtained from the Langmuir model fitting are 817.22, 879.42, 1456.28, and 1248.31 mg g^−1^, respectively, all of which are higher than the experimental maximum adsorption capacities (q_m,Exp_ = 529.23, 535.19, 647.91, and 660.56 mg g^−1^). This may be attributable to the fact that the experimental concentration range (200–500 mg L^−1^) does not fully cover the adsorption saturation plateau, and the theoretical monolayer saturation capacity predicted by the Langmuir model exceeds the practically accessible capacity. The Langmuir equilibrium constants (k_L_) are 0.0040, 0.0034, 0.0018, and 0.0025 L mg^−1^, respectively, and the corresponding dimensionless separation factors (R_L_ = 1/(1 + k_L_·c_0_)) range from 0.33 to 0.85, all falling between 0 and 1, indicating that the adsorption of CR onto UNA-Tt is a favourable adsorption process.

Figure 5b presents the linear fitting plots of the Freundlich model (ln q_e_ vs. ln c_e_). The fitting curves of all samples exhibit a relatively good linear trend. The Freundlich constants 1/n are 0.478, 0.526, 0.680, and 0.601, respectively, all falling between 0 and 1, indicating that the adsorption process is favourable. Moreover, the 1/n values of UNA-T45 and UNA-T60 are closer to 0.5, suggesting that their surface energy distributions are relatively homogeneous and that the interaction intensity between the adsorbate and the adsorbent surface is moderate. However, the R^2^ values of the Freundlich model (0.86685–0.92686) are all lower than those of the Langmuir and Temkin models, indicating that this model provides relatively limited accuracy in describing the adsorption behaviour of CR onto UNA-Tt.

Figure 5c presents the linear fitting plots of the Temkin model (q_e_ vs. ln c_e_). The fitting curves of all samples exhibit excellent linear correlations, with R^2^ values of 0.96376, 0.95983, 0.98358, and 0.97904, respectively, which are the highest among the three models. The Temkin constants B (a parameter related to the heat of adsorption) are 276.98, 289.51, 352.93, and 349.51 J mol^−1^, respectively, showing an increasing trend with prolonged sonication time, indicating that UNA-T45 and UNA-T60 possess higher adsorption heats and stronger interactions between the adsorbate and the adsorbent surface. The Temkin equilibrium constants A are 0.02, 0.01, 0.01, and 0.02 L g^−1^, respectively. Based on the fitting performance of the three models, the Temkin model most accurately describes the adsorption behaviour of CR onto UNA-Tt, indicating that during the adsorption process, the heat of adsorption decreases linearly with increasing surface coverage, and that there is a significant interaction between the adsorbate and the adsorbent.

**Figure 6:**
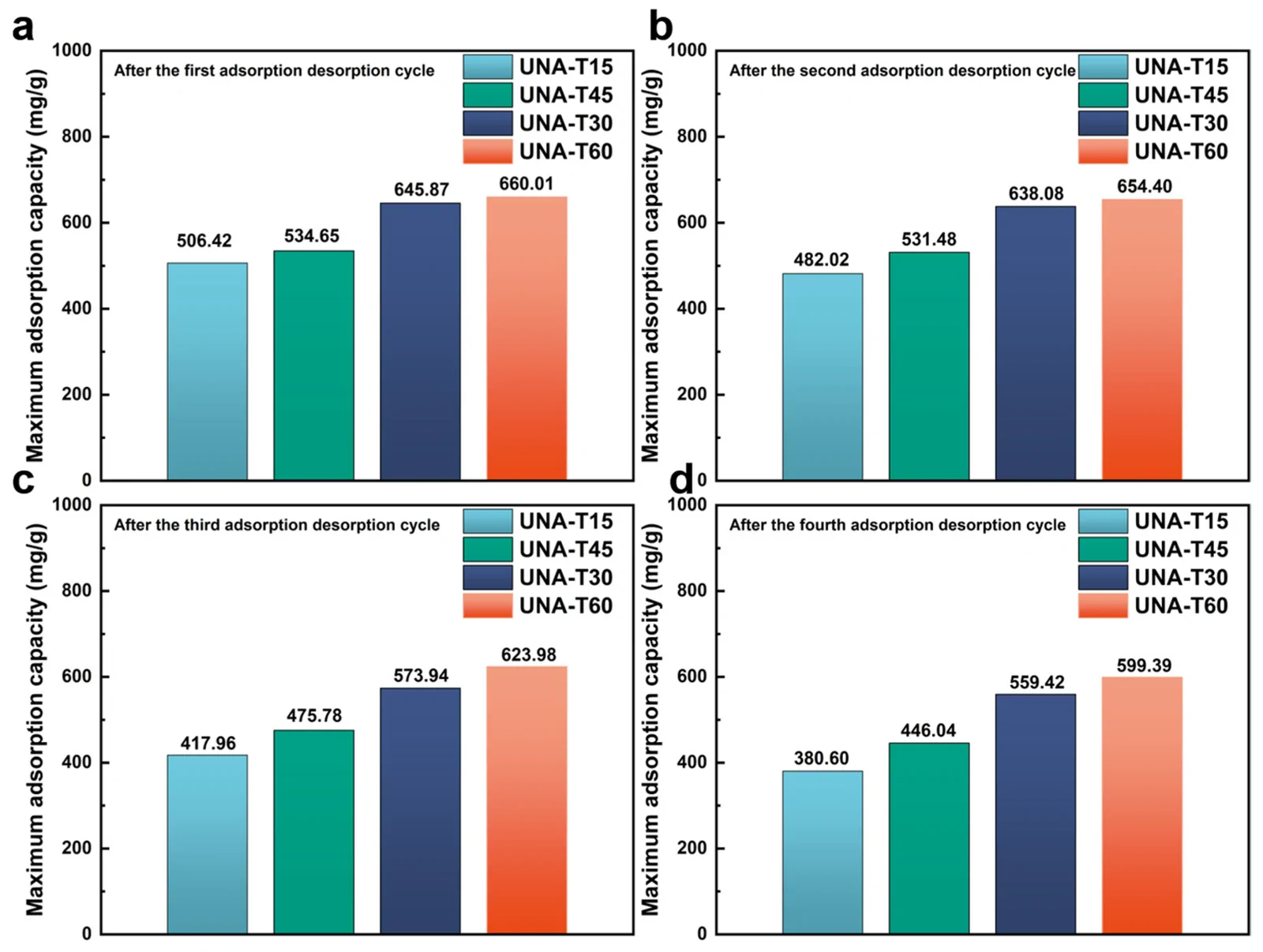
UNA-Tt for CR at 500 mg L^−1^: (**a**) maximum adsorption capacity after the first regeneration cycle, (**b**) maximum adsorption capacity after the second regeneration cycle, (**c**) maximum adsorption capacity after the third regeneration cycle, and (**d**) maximum adsorption capacity after the fourth regeneration cycle.

To evaluate the practical application potential of the UNA-Tt series samples, four consecutive adsorption–desorption cyclic regeneration experiments were conducted, with the results shown in Figure 6. Figure 6a presents the maximum adsorption capacities after the first regeneration cycle. The maximum adsorption capacities of UNA-T15, UNA-T30, UNA-T45, and UNA-T60 are 506.42, 534.65, 645.87, and 660.01 mg g^−1^, respectively, which are close to those of the fresh samples (529.23, 535.19, 647.91, and 660.56 mg g^−1^), indicating that the regeneration process causes relatively little damage to the material structure. Figure 6b shows the maximum adsorption capacities after the second regeneration cycle. The adsorption capacities of each sample decrease to 482.02, 531.48, 638.08, and 654.40 mg g^−1^, respectively, with UNA-T15 exhibiting the most pronounced capacity (a decrease of 4.8%), while the capacity retention rates of UNA-T45 and UNA-T60 remain higher than 98.8%. Figure 6c presents the maximum adsorption capacities after the third regeneration cycle. The capacities further decrease to 417.96, 475.78, 573.94, and 623.98 mg g^−1^, with cumulative decreases of 17.5% for UNA-T15, 11.0% for UNA-T30, 11.0% for UNA-T45, and 5.5% for UNA-T60. Figure 6d shows the maximum adsorption capacities after the fourth regeneration cycle. The adsorption capacities of the samples are 380.60, 446.04, 559.42, and 599.39 mg g^−1^, respectively, with UNA-T60 exhibiting the highest capacity retention rate (90.8%), which is significantly superior to those of UNA-T15 (75.2%), UNA-T30 (83.4%), and UNA-T45 (86.6%).

The exceptional cyclic stability of UNA-T60 (90.8% retention after four cycles) is attributed to its highly crystalline, regular octahedral framework structure, as evidenced by the sharp XRD peaks and well-defined crystal morphology (Figure 1 and Figure 2c). The robust intercrystalline contacts within the self-assembled aerogel resist structural collapse during repeated regeneration. In contrast, the inferior stability of UNA-T15 (75.2% retention) correlates with its low-crystallinity, amorphous aggregate morphology (Figure S1), which is more susceptible to mechanical degradation during ultrasonic regeneration. Thus, crystallinity and structural integrity—directly controlled by sonication time—are identified as the key factors governing reusability.

## 3. Conclusions

In summary, we have successfully developed a green, rapid, and scalable strategy for the ambient-temperature construction of micro-/mesoporous UiO-66-NH_2_ self-assembled aerogels, in which the critical MOF crystallization and in situ self-assembly stages are driven solely by ultrasound at room temperature. This approach fundamentally overcomes the key engineering bottlenecks of conventional MOF powders-namely, poor recyclability and high mass-transfer resistance-by achieving in situ nanocrystal self-assembly into a monolithic, self-supporting framework. Critically, the MOF crystallization and aerogel assembly stages proceed under ambient temperature and pressure, thereby avoiding the prolonged high-temperature solvothermal conditions typically required for UiO-66-NH_2_ synthesis, as well as toxic solvents and polymeric binders, thus aligning with the principles of sustainable materials chemistry.

Our systematic investigation suggests that ultrasonic cavitation may play a pivotal role: it appears to facilitate both the reduction in the nucleation energy barrier for rapid crystallization and the creation of conditions favorable for nanocrystal assembly into an interconnected three-dimensional network. The sonication time was identified as a key parameter associated with structural evolution, with the optimal UNA-T60 sample exhibiting fully developed regular octahedral crystals, a high specific surface area (1196.9 m^2^ g^−1^), and a well-balanced bimodal micro-/mesoporous architecture. This bimodal micro-/mesoporous hierarchy is the cornerstone of the material’s exceptional performance: micropores furnish abundant adsorption sites while mesopores serve as efficient molecular transport corridors, synergistically enhancing both adsorption capacity and kinetics.

The resulting UNA-T60 aerogel demonstrated outstanding CR removal capability, achieving a maximum adsorption capacity of 660.56 mg g^−1^ with rapid kinetics that conform to the pseudo-second-order model. This kinetic behavior is consistent with a surface adsorption-limited process governed by the hierarchical micro-/mesoporous architecture, wherein rapid mass transfer through mesoporous channels facilitates efficient occupation of microporous adsorption sites. No specific chemical interaction mechanism is assigned in this study, as such conclusions would require dedicated spectroscopic evidence beyond the scope of the present characterization. The robust monolithic structure and high specific surface area endowed the aerogel with excellent reusability, maintaining 90.8% of its initial capacity after four regeneration cycles—a significant advantage over its powder counterparts. The Temkin isotherm model provided the best fit for the adsorption data, suggesting a linear decrease in adsorption heat with surface coverage and confirming significant adsorbate–adsorbent interactions.

This work presents a transformative methodology for the green synthesis of pure MOF aerogels and establishes a clear structure-performance relationship based on pore architecture and specific surface area. The successful demonstration of this strategy opens new avenues for the rational design of micro-/mesoporous adsorbents and their practical application in continuous-flow wastewater treatment systems. We acknowledge that a notable limitation of the present study is the lack of replicate experiments and statistical error analysis in the adsorption measurements, which restricts the rigorous quantitative assessment of result reproducibility. Future work will incorporate systematic replicate measurements and comprehensive statistical treatment. Additionally, future research will focus on extending this ultrasound-assisted assembly approach to other MOF systems and exploring the influence of ultrasonic frequency and power on the fine-tuning of aerogel architectures for targeted pollutant removal.

## 4. Experimental Materials and Methods

### 4.1. Materials

Accordingly, 2-Aminoterephthalic acid (H_2_BDC-NH_2_, 98%), zirconium n-propoxide (C_12_H_28_O_4_Zr, 70wt%), N,N’-dimethylformamide (DMF, 99.5%), glacial acetic acid (HAc, 99.5%), and Congo Red (CR, 99%) were used as received. All other chemical reagents were of analytical grade and used without further purification.

### 4.2. Material Preparation

Preparation of UNA-Tt Aerogels: The synthesis procedure consists of two distinct stages. Stage I (Precursor Activation): Zirconium n-propoxide (821 μL, 1.83 mmol, 70 wt% in n-propanol), glacial acetic acid (24 mL, 0.42 mol), and DMF (42 mL) were placed in a polytetrafluoroethylene-lined stainless-steel autoclave and reacted at 130 °C for 3 h to obtain the pre-hydrolyzed zirconium source activated solution. It is emphasized that this thermal step serves exclusively for precursor hydrolysis and activation; no MOF crystallization occurs under these conditions. Stage II (Ambient-Temperature MOF Crystallization and Aerogel Assembly): All subsequent MOF nucleation, crystallization, and gelation proceed at room temperature (~25 °C) under ultrasonic irradiation. After the activated solution was cooled to ambient temperature, 2-aminoterephthalic acid (H_2_BDC-NH_2_, 0.3318 g, 1.83 mmol) was added, and the mixture was subjected to ultrasonic dispersion for 10 min to ensure complete dissolution. Subsequently, the mixed solution was transferred to an open glass reaction vessel and placed in an ultrasonic cleaner, where the ultrasonic reaction was conducted at a power of 900 W. By regulating the sonication time (15, 30, 45, and 60 min), the UNA-T15, UNA-T30, UNA-T45, and UNA-T60 samples were obtained, respectively. Upon completion of the reaction, the products were centrifuged (9000 r min^−1^, 10 min), the supernatant was discarded, and the precipitate was redispersed in anhydrous ethanol with additional sonication for 10 min. This centrifugation–washing cycle was repeated three times to remove residual solvents and unreacted precursors. The final products were stored at −5 °C for 12 h and then vacuum-dried at 60 °C for 12 h to thoroughly eliminate residual solvent molecules, yielding the UiO-66-NH2 self-assembled aerogels. The detailed synthesis parameters for each sample are provided in Table 2.

Cyclic Regeneration Experiments of UNA-Tt: The CR-saturated UNA-Tt aerogels were separated from the dye solution by filtration and thoroughly washed with deionized water. The washed aerogels were then immersed in fresh deionized water and subjected to ultrasonic treatment for 15 min to facilitate the desorption of residual dyes from the surface and pore channels. The resulting dispersion was centrifuged (9000 r min^−1^, 10 min), and the dye-containing supernatant was discarded. This water-washing–ultrasonication–centrifugation cycle was repeated three times. To further remove organic residues and restore the pore structure, the obtained solids were immersed in anhydrous ethanol and subjected to ultrasonic treatment for 15 min, followed by centrifugal separation. This ethanol-washing–ultrasonication–centrifugation cycle was also repeated three times. Finally, the regenerated aerogels were placed in a vacuum oven at 120 °C for 3 h to thoroughly remove residual solvents. After cooling to ambient temperature, the regenerated materials were employed for the subsequent round of adsorption testing.

### 4.3. Characterization Test Methods

The structural characteristics of UNA-Tt were systematically analyzed using multiscale characterization techniques. The crystal structure was determined using a Bruker D8 Advance X-ray diffractometer (Bruker Corporation, Billerica, MA, USA) under the following conditions: Cu Kα radiation (λ = 1.54182 Å), operating voltage of 30 kV, scanning range of 5°–90°, and scanning rate of 2°·min^−1^. The specific surface area and pore structure parameters were measured using a Quantachrome Autosorb iQ MP (Quantachrome Instruments, Boynton Beach, FL, USA) fully automated surface area and porosity analyzer. The samples were pretreated by vacuum degassing at 120 °C for 5 h, and high-purity nitrogen was employed as the adsorbate for adsorption–desorption measurements at 77 K. The surface functional groups and chemical bond vibration information were characterized using a Bruker Vertex 80v Fourier-transform infrared spectrometer (Bruker Corporation, Billerica, MA, USA). The samples were mixed with KBr at a mass ratio of approximately 1:100 and pressed into pellets, with the measurement range spanning 400–4000 cm^−1^. The microstructural morphology was examined using a Zeiss Sigma 300 field-emission scanning electron microscope (Carl Zeiss AG, Oberkochen, Germany) and a Thermo Scientific Talos F200X transmission electron microscope (Thermo Fisher Scientific, Waltham, MA, USA). For SEM analysis, the samples were mounted on conductive adhesive tape, sputter-coated with gold for 120 s, and observed at an accelerating voltage of 3–5 kV.

### 4.4. Study on Storage and Transport Rate of Anionic Dyes

All adsorption experiments were conducted at ambient temperature. Accurately weighed 5 mg of the as-prepared UNA-Tt sample was employed as the adsorbent and added to 20 mL of CR dye solutions with varying initial mass concentrations. It should be noted that, due to time constraints during the preliminary experimental phase, replicate measurements were not systematically performed; the reported adsorption capacities represent single determinations under the stated conditions. The mixed suspensions were subjected to ultrasonic treatment in an ultrasonic cleaner for 10 min, followed by static standing for 5 min. The supernatant was then collected, and its absorbance was measured using a UV–visible spectrophotometer (Nanjing Kejie Analytical Instrument Co., Ltd., Nanjing, China) at the maximum absorption wavelength. The residual dye concentration was calculated based on the standard calibration curve. This procedure was repeated until adsorption equilibrium was attained.

#### 4.4.1. Adsorption Capacity

The adsorption capacity, q_e_(mg g^−1^), is defined as the amount of adsorbate adsorbed per unit mass of adsorbent at a given temperature and adsorbate concentration. The dye concentration in solution was determined by UV–visible spectrophotometry, and the adsorption capacity of the adsorbent for the dye molecules was calculated according to Equation (1):

(1) $$q_{e}=\frac{\left(c_{0}-c_{e}\right)}{m}V$$

where c_0_ and c_e_ denote the initial and equilibrium concentrations of the dye solution (mg·L^−1^), respectively; V represents the volume of the dye solution (L); m is the mass of the adsorbent (g); and q_e_ is the adsorption capacity of the adsorbent for the dye molecules at equilibrium (mg g^−1^).

#### 4.4.2. Adsorption Kinetics

The kinetic study of the adsorption process is primarily employed to describe the rate at which the adsorbent captures the adsorbate. Kinetic models are fitted to the experimental data to elucidate the underlying adsorption mechanisms. The pseudo-first-order kinetic model assumes that the adsorption rate is directly proportional to the number of unoccupied adsorption sites. The pseudo-second-order kinetic model posits that the adsorption rate is proportional to the square of the number of unoccupied adsorption sites, reflecting a surface adsorption-controlled process. The intraparticle diffusion (Weber–Morris) model is utilized to identify the rate-controlling step and that two distinct binding sites are present on the adsorbent surface. The intraparticle diffusion (Weber–Morris) model is utilized to identify the rate-controlling step of the physicochemical reaction. The adsorption process is divided into two sequential stages: rapid surface adsorption onto the adsorbent and slow diffusion within the pore channels. The mathematical expressions for the pseudo-first-order, pseudo-second-order, and intraparticle diffusion models are given by Equations (2)–(4), respectively:

(2) $$\ln\left(q_{e}-q_{t}\right)=lnq_{e}-k_{1}t$$

(3) $$\frac{t}{q_{t}}=\frac{1}{k_{2}q_{e}^{2}}+\frac{t}{q_{e}}$$

(4) $$q_{t}=k_{p}t^{0.5}+I$$

where q_e_ and q_t_ represent the adsorption capacities at equilibrium and at time t (mg·g^−1^), respectively; t is the adsorption time (min); I is a parameter; and k_1_ (min^−1^), k_2_ (g mg^−1^ min^−1^), and k_p_ (mg g^−1^ min^−0.5^) are the rate constants for the pseudo-first-order, pseudo-second-order, and intraparticle diffusion models, respectively.

#### 4.4.3. Isothermal Adsorption Model

(1) Langmuir’s isothermal adsorption model

The Langmuir model is one of the commonly used adsorption models, which assumes that adsorption occurs uniformly on all active sites of the adsorbent, and the adsorbate undergoes monolayer adsorption on the surface of the adsorbent. The Langmuir model assumes monolayer adsorption on a homogeneous surface with a finite number of identical adsorption sites. The linear expression of the Langmuir model is shown in formula (5):

(5) $$\frac{C_{e}}{q_{e}}=\frac{C_{e}}{q_{m}}+\frac{1}{k_{L}q_{m}}$$

where c_e_ is the equilibrium concentration (mg L^−1^); q_m_ is the saturated adsorption capacity (mg g^−1^); q_e_ is the equilibrium adsorption capacity (mg g^−1^); and K_L_ is the adsorption equilibrium constant (L mg^−1^).

(2) Freundlich’s isothermal adsorption model

The Freundlich model is predicated on the assumption that the adsorbent surface possesses heterogeneous energy distribution, and that the target species undergoes multilayer adsorption on the adsorbent surface rather than a single monolayer. The mathematical expression of the Freundlich isotherm model is given by Equation (6):

(6) $$lnq_{e}=lnK_{F}+\frac{1}{n}lnc_{e}$$

where c_e_ is the equilibrium concentration (mg L^−1^); q_e_ is the equilibrium adsorption capacity (mg g^−1^); K_F_ is the Freundlich equilibrium constant [(mg g^−1^)(L mg^−1^)^1/n^]; and *n* is the Freundlich equilibrium constant representing the adsorption intensity.

(3) Temkin’s isothermal adsorption model

The Temkin model postulates that the heat of adsorption of the adsorbate on the surface decreases linearly with increasing surface coverage. The mathematical expression of the Temkin isotherm model is given by Equation (7):

(7) $$q_{e}=Bln\left(A\right)+Blnc_{e}$$

where q_e_ is the equilibrium adsorption capacity (mg g^−1^); c_e_ is the equilibrium concentration (mg L^−1^); *B* is a parameter related to the heat of adsorption; and *A* is the equilibrium constant of the Temkin model (L g^−1^).

## Figures and Tables

**Table 1:** Pore structure information of UNA-Tt series samples.

	S_BET_/( m^2^ g^−1^)	S_Langmuir_/(m^2^ g^−1^)	V_pore_/(cm^−3^ g^−1^)	Proportion of Micropores/(%)	Proportion of Mesopores/(%)
UNA-T15	950.7	1435.7	0.6375	52.1	47.9
UNA-T30	957.7	1739.5	0.9082	28.5	71.5
UNA-T45	1127.6	1517.7	0.6672	58.7	41.3
UNA-T60	1196.9	1656.8	0.7166	57.2	42.8

**Table 2:** Experimental formula table of UNA-Tt.

Sample	Zirconium Source	Ligand	Synthesis Temperature	Synthesis Time	Ultrasonic Power
UNA-T15	C_12_H_28_O_4_Zr	H_2_BDC-NH_2_	room temperature	15 min	900 W
UNA-T30	C_12_H_28_O_4_Zr	H_2_BDC-NH_2_	room temperature	30 min	900 W
UNA-T45	C_12_H_28_O_4_Zr	H_2_BDC-NH_2_	room temperature	45 min	900 W
UNA-T60	C_12_H_28_O_4_Zr	H_2_BDC-NH_2_	room temperature	60 min	900 W

## Data Availability

The data presented in this study are openly available in article.

## Supplementary Materials

The following supporting information can be downloaded at https://www.mdpi.com/article/10.3390/gels12090778/s1. Figure S1. Scanning electron microscopy (SEM) images of hierarchically porous UiO-66-NH_2_ self-assembled aerogels at various magnifications. (**a**–**c**) UNA-T15; (**d**–**f**) UNA-T30; (**g**–**i**) UNA-T45; (**j**–**l**) UNA-T60. Each row corresponds to progressively increasing magnifications, enabling a comparative analysis of the microstructural morphology and pore architecture evolution of the aerogels as a function of sonication time. Figure S2. Nonlinear regression fitting of the pseudo-first-order (PFO, dashed red curves) and pseudo-second-order (PSO, solid blue curves) models to the experimental kinetic data (symbols) for CR adsorption onto (**a**) UNA-T15, (**b**) UNA-T30, (**c**) UNA-T45, and (**d**) UNA-T60. Table S1: Fitting Parameters of UNA-Tt. Table S2: Performance Comparison of Dye Adsorbents for Congo Red Adsorption [19,47,48,49,50].
