# Supplementary material for: Ultrasound-Induced In Situ Self-Assembly of Bimodal Micro/Mesoporous UiO-66-NH2 Aerogels for High-Performance Congo Red Capture
Source: Gels. 2026 Aug 31;12(9):778. doi: 10.3390/gels12090778 (PMC13606375; doi:10.3390/gels12090778)
Supplement: Supplementary file 1 [file gels-12-00778-s001.zip › gels-4487836-supplementary.pdf]

Ultrasound-Induced In Situ Self-Assembly of Bimodal Micro-/Mesoporous UiO-66-NH_2 Aerogels for High-Performance Congo Red Capture

Tian Zhao *, Shilin Peng, Yan Wu, Tianhang Wang, Xing Zhang, Zhuoheng Li, Xiangjiang Wu, Ying Chen and Yi Chen *

School of Materials Science and Engineering, Hunan University of Technology, Zhuzhou, Hunan 412007

* Correspondence: tian_zhao@hut.edu.cn (T. Zhao), chenyi@hut.edu.cn (Y. Chen)

Abstract

Metal–organic framework (MOF) powders possess remarkable adsorption capabilities, yet their practical application is severely hampered by poor processability, difficult recovery, and high mass-transfer resistance. Here, we report a green and rapid two-stage strategy for constructing UiO-66-NH_2 self-assembled aerogels with a bimodal micro-/mesoporous architecture. A brief thermal pretreatment (130 °C, 3 h) is used solely for precursor activation, after which the critical MOF crystallization and in-situ self-assembly are driven under ambient conditions via ultrasonic cavitation (900 W, 15-60 min). This protocol simultaneously drives the nucleation, crystallization, and self-assembly of UiO-66-NH_2 nanocrystals into a monolithic, self-supporting architecture, thereby replacing the conventional, prolonged, high-temperature solvothermal MOF crystallization with a rapid, room-temperature process. The sonication time critically governs the structural evolution, transforming initially amorphous aggregates into well-defined regular octahedral nanocrystals that form an interconnected framework. The optimized aerogel (UNA-T60) exhibits an exceptional specific surface area (1196.9 m^2 g^{-1}) and a synergistic bimodal pore structure comprising intrinsic micropores and intercrystalline mesopores. This architecture enables rapid mass transfer and active-site accessibility, resulting in a maximum Congo Red (CR) adsorption capacity of 660.56 mg g^{-1}, with kinetics conforming to the pseudo-second-order model ($R^2 > 0.999$). The robust monolithic structure endows the material with outstanding reusability, retaining 90.8 % of its initial adsorption capacity after four regeneration cycles. This work presents a paradigm-shifting approach for the sustainable fabrication of pure MOF aerogels, offering a promising solution for advanced dye wastewater treatment.

Keywords: metal-organic framework aerogels; ultrasound-assisted synthesis; hierarchical micro-/mesopores; Congo Red adsorption

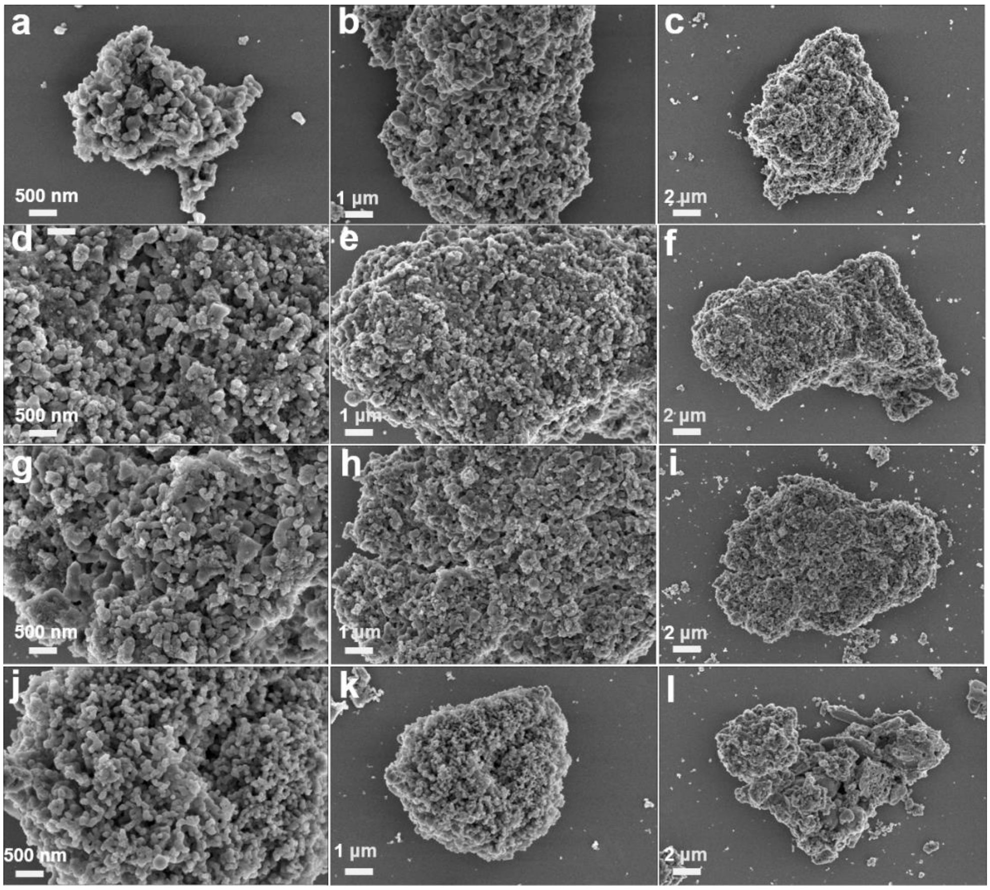

Figure S1. Scanning electron microscopy (SEM) images of hierarchically porous UiO-66-NH_2 self-assembled aerogels at various magnifications. (a–c) UNA-T15; (d–f) UNA-T30; (g–i) UNA-T45; (j–l) UNA-T60. Each row corresponds to progressively increasing magnifications, enabling a comparative analysis of the microstructural morphology and pore architecture evolution of the aerogels as a function of sonication time.

The microstructural evolution of the UNA-Tt series samples synthesized under varying sonication times was systematically investigated by scanning electron microscopy (SEM), with the results presented in Fig S1. Fig. S1a–c display SEM images of UNA-T15 (sonicated for 15 min) at different magnifications: the low-magnification view (Fig. S1c, scale bar 2 μm) reveals irregular block-like aggregates with indistinct boundaries, while the high-magnification image (Fig. S1a, scale bar 500 nm) further elucidates that the surface is densely packed with numerous amorphous particles, with no discernible crystal facet development. This indicates that the energy input provided by the relatively short sonication duration (15 min) is insufficient to

overcome the nucleation energy barrier of UiO-66-NH_2, resulting in incomplete crystal growth. Consequently, the precursors predominantly exist as low-crystallinity intermediates, leading to severe amorphous aggregation.

When the sonication time was extended to 30 min (UNA-T30, Fig. S1d–f), a pronounced morphological transformation was observed. The high-magnification image (Fig. S1d, scale bar 500 nm) reveals that the nanocrystallite dimensions increased with progressively sharper contours, and the intercrystalline packing became more loosely arranged, giving rise to distinct mesoporous channels. The medium-magnification view (Fig. S1e; scale bar, 1 μm) demonstrates an enhanced surface roughness of the aggregates, exhibiting characteristic features of nanocrystal assembly. The low-magnification image (Fig. S1f; scale bar, 2 μm) further indicates that the block-like morphology became more regular with sharpened edges. These observations confirm that the extended sonication time effectively facilitated further crystal nucleus growth and interfacial self-assembly. The substantial mesoporosity generated by the disordered packing of nanocrystals provided a preliminary pore network for subsequent mass transfer; however, the typical regular octahedral crystal morphology characteristic of UiO-66-NH_2 had not yet emerged.

After 45 minutes of ultrasound (UNA-T45, Fig. S1g-i), the degree of crystal development significantly increased. The high magnification image (Fig. S1g, scale 500 nm) can identify some well-developed polyhedral nanocrystals (particle size about 50-100 nm), and the crystal surface characteristics are initially apparent; The medium magnification field of view (Fig. S1h, scale 1 μm) shows that these nanocrystals are cross-linked with each other, forming a three-dimensional porous skeleton; The low magnification image (Fig. S1i, scale 2 μm) indicates that the aggregates maintain a block like morphology as a whole, but the surface porosity increases significantly. At this stage, the coexistence of octahedral crystals and an amorphous matrix reflects that the nucleation and growth process has become more complete, but the ultrasound energy is not yet sufficient to drive all grains to complete orientation growth and dense assembly.

When the ultrasound time was extended to 60 minutes (UNA-T60, Fig. S1j-l), the

sample exhibited typical hierarchical porous structure characteristics. The high magnification image (Fig. S1j, scale 500 nm) clearly displays a large number of uniformly sized regular octahedral nanocrystals (particle size about 80-120 nm), with well-developed crystal faces and sharp edges. The grains are assembled with each other through point or surface contact, forming rich intergranular pores; In the medium magnification field of view (Figure S1k, scale 1 μm), it can be seen that the nanocrystalline assembly has a loose and porous sponge like structure; The low power image (Fig. S1l, scale 2 μm) shows that the aerogel is loosely overlapped by several micron aggregates, and the macro pores between aggregates and the meso pores between grains are interconnected. It is worth noting that although macroscopic voids (>50 nm) between aggregates can be observed in the low magnification field of SEM, BET test results show that the pore structure of UNA-T60 is mainly composed of micropores (<2 nm, originating from UiO-66-NH_2 intrinsic pores) and mesopores (2-50 nm, originating from the stacking gaps between nanocrystals), and no significant contribution of macropores was detected. This phenomenon may be attributed to (1) partial shrinkage of the gel skeleton during freeze-drying leads to macropore collapse; (2) the proportion of macropore volume is too low, exceeding the sensitivity range of the BET nitrogen adsorption–desorption test; (3) the gaps between aggregates partially closed during the vacuum degassing pretreatment process at 120 °C. Based on BET quantitative results, the pore structure of UNA-T60 can be clearly defined as a dual-stage hierarchical system of micropores and mesopores: micropores provide abundant surface adsorption sites, while mesopores act as molecular transport channels, significantly reducing mass transfer resistance. This dual-stage, synergistic structure endows UNA-T60 with excellent adsorption kinetics and capacity advantages.

The above morphological evolution patterns indicate that ultrasound time plays a decisive regulatory role in the nucleation, growth, and self-assembly processes of UiO-66-NH_2 nanocrystals. The localized high-temperature and high-pressure microenvironment generated by ultrasonic cavitation effectively reduces the nucleation energy barrier of crystals and promotes the rapid generation of crystal nuclei; At the same time, the cavitation microjet and turbulent disturbance enhance the random

collision and aggregation between nanocrystals, providing a unique dynamic window for the in-situ generation of mesopores and macropores. With the extension of ultrasonic time, nanocrystals gradually developed into complete octahedra, and a hierarchical aerogel framework with a self-assembled pore structure was formed.

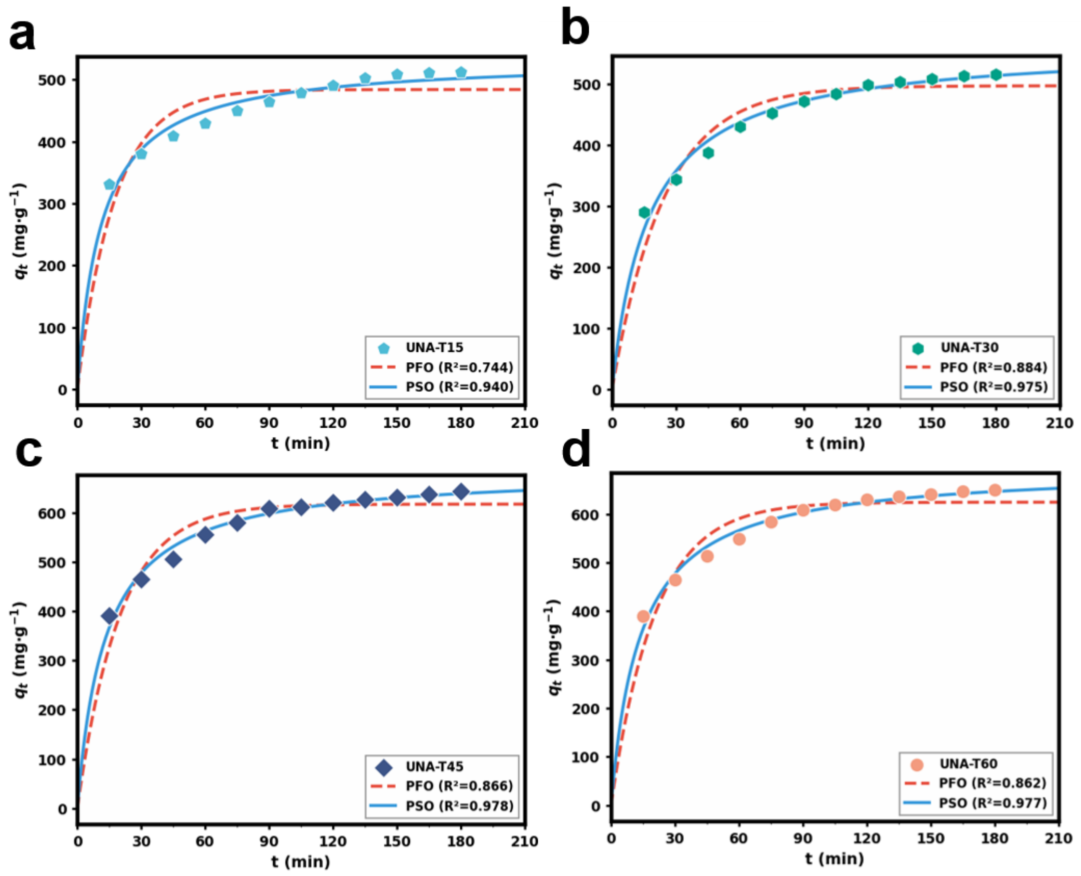

Figure S2. Nonlinear regression fitting of the pseudo-first-order (PFO, dashed red curves) and pseudo-second-order (PSO, solid blue curves) models to the experimental kinetic data (symbols) for CR adsorption onto (a) UNA-T15, (b) UNA-T30, (c) UNA-T45, and (d) UNA-T60.

Fitting was performed on the untransformed data using the Levenberg–Marquardt algorithm in OriginPro 2024 to avoid distortion of errors associated with linearization. The corresponding nonlinear regression parameters (R^2, RMSE, AIC, BIC, and standard errors) are summarized in Table S1.

Table S1. Fitting Parameters of UNA-Tt.

		Parameter	UNA-T15	UNA-T30	UNA-T45	UNA-T60
Mass Transfer Kinetics	Weber - Morris	K_{p1}(mg g^{-1} t$^{0.5}$)	22.04	30.9	33.20	34.27
		I_1（mg g^{-1}）	255.17	182.86	280.61	274.48
		R_1^2	0.98923	0.98313	0.96613	0.97780
		K_{p2}(mg g^{-1} t$^{0.5}$)	5.22	7.08	9.15	8.52
		I_2（mg g^{-1}）	443.00	422.13	520.00	536.59
		R_2^2	0.87202	0.97251	0.99928	0.99264
Adsorption Kinetics	PFO (linear)	$q_{e,Exp}$ (mg g^{-1})	511.94	516.25	642.97	650.57
		$q_{e,Cal}$ (mg g^{-1})	338.30	389.98	396.49	359.67
		k_1 (min^{-1})	0.02394	0.02442	0.02423	0.02063
		R^2	0.97296	0.99186	0.97115	0.99707
	PFO (nonlinear)	$q_{e,Cal}$ (mg g^{-1})	484.09	497.49	617.36	624.70
		k_1 (min^{-1})	0.0572	0.0409	0.0503	0.0485
		R^2	0.7441	0.8836	0.8657	0.8622
		*R*MSE (mg g^{-1})	28.15	24.14	28.00	29.50
		AIC	84.10	80.42	83.97	85.22
	PSO (linear)	$q_{e,Exp}$ (mg g^{-1})	511.94	516.25	642.97	650.57
		$q_{e,Cal}$ (mg g^{-1})	552.49	571.43	689.66	704.23
		k_2 (g mg^{-1} min^{-1})	0.000123	0.000094	0.000140	0.000095
		R^2	0.99845	0.99907	0.9995	0.99944
	PSO (nonlinear)	$q_{e,Cal}$ (mg g^{-1})	533.74	563.40	685.03	695.99
		k_2 (g mg^{-1} min^{-1})	0.000165	0.000103	0.000112	0.000105
		R^2	0.9401	0.9745	0.9777	0.9772
		*R*MSE (mg g^{-1})	13.61	11.30	11.42	12.01
		AIC	66.67	62.20	62.44	63.65
Adsorption Isotherm Models	Langmuir	$q_{m,Exp}$ (mg g^{-1})	529.23	535.19	647.97	660.56
		$q_{m,Cal}$ (mg g^{-1})	817.22	879.42	1456.28	1248.31
		k_L (L mg^{-1})	0.0040	0.0034	0.0018	0.0025

	K'	0.33-0.72	0.37-0.75	0.53-0.85	0.45-0.80
	R^2	0.96177	0.90890	0.81978	0.88701
	1/n	0.47766	0.52567	0.67972	0.60056
Freundlich	k_F (mg g^{-1} (L $mg^{-1})^{1/n}$)	28.64	21.66	10.26	17.02
	R^2	0.91474	0.86685	0.92686	0.91408
	B	276.98	289.51	352.93	349.51
Temkin	A (L g^{-1})	0.02	0.01	0.01	0.02
	R^2	0.96376	0.95983	0.98358	0.97904

Table S2. Performance Comparison of Dye Adsorbents for Congo Red Adsorption.

Material	Maximum adsorption capacity (mg/g)	Adsorption capacity retention rate after four cycles(%)	References
UNA-T60	660.56	90.8	This work
UNA-T45	647.91	86.6	This work
UNA-T60	535.19	83.4	This work
UNA-T15	529.23	75.2	This work
Al-MOF	80.64	65	[47]
Al-MOF@C	68.96	> 60	[47]
Mesoporous UiO-66(U)	144.9	N/A	[48]
UiO-66@β-CD@Fe_3O_4	542.75	N/A	[49]
In situ UiO-66-NH_2/TOCNF aerogel	549.45	N/A	[19]
UiO-66-NH_2@SiO_2	393.83	N/A	[50]

N/A in the table represents Not Available.
